# Broad Expression Analysis of Human ANTXR1/TEM8 Transcripts Reveals Differential Expression and Novel Splizce Variants

**DOI:** 10.1371/journal.pone.0043174

**Published:** 2012-08-17

**Authors:** Micaela Vargas, Raghavendra Karamsetty, Stephen H. Leppla, G. Jilani Chaudry

**Affiliations:** 1 Cell and Molecular Biology Program, Department of Biology, The University of Texas at San Antonio, San Antonio, Texas, United States of America; 2 Microbial Pathogenesis Section, The Laboratory of Parasitic Diseases, National Institute of Allergy and Infectious Diseases, National Institutes of Health, Bethesda, Maryland, United States of America; Institute of Molecular and Cell Biology, Singapore

## Abstract

Tumor endothelial marker 8 (TEM8; ANTXR1) is one of two anthrax toxin receptors; the other is capillary morphogenesis gene 2 protein (CMG2; ANTXR2). TEM8 shows enhanced expression in certain tumor endothelia, and is thought to be a player in tumor vasculature formation. However, a comprehensive expression profile of individual TEM8 variants in normal or cancerous tissues is lacking. In this work we carried out an extensive analysis of all splice variants of human TEM8 in 12 digestive tissues, and 8 each fetal and adult tissues, 6 of them cognate pairs. Using variant-specific primers, we first ascertained the status of full-length transcripts by nested PCR. We then carried out quantitative analysis of each transcript by real-time PCR. Three splice variants of TEM8 were reported before, two single-pass integral membrane forms (V1 and V2) and one secreted (V3). Our analysis has revealed two new variants, one encoding a membrane-bound form of the receptor and the other secreted, which we have designated V4 and V5, respectively. All tissues had V1, V2, V3, and V4, but only prostate had V5. Real-time PCR revealed that all variants are present at different levels in various tissues. V3 appeared the most abundant of all. To ascertain its functionality for anthrax toxin, we expressed the newly identified form V4 in a receptor-negative host cell, and included V1 and V2 for comparison. Cytotoxicity, toxin binding, and internalization assays showed V4 to be as efficient a receptor as V1 and V2.

## Introduction

Tumor endothelial marker 8 (TEM8) was originally discovered by serial analysis of gene expression (SAGE) in endothelial cells of colon carcinomas [1]. The analysis revealed markedly elevated levels of TEM8 mRNA in these cells, and normal endothelial cells appeared to have little of it. Further, these studies identified three splice variants of the gene, V1, V2, and V3. The variants encode proteins of 564, 368, and 333 residues, respectively. Topologically, V1 and V2 are type-1 integral membrane proteins with a single tramsmembrane helix. V2 is identical to V1 up to residue 364, but the last four residues of V2 are unique, a consequence of differential splicing. V3 sequence diverges from V1 and V2 right before the beginning of the transmembrane helix. Thus, V3 has a portion that is identical to the extracellular portions of V1 and V2, but its 13-residue carboxyl terminal segment is unique.

Using a human cDNA library for expression cloning, Bradley et al. [2] independently identified TEM8 V2 as an anthrax toxin receptor. Later studies showed that V1 also functions as an anthrax toxin receptor, and that V3 does not [3]. Thus, these findings were consistent with the original reports that V1 and V2 are integral membrane proteins and V3 is a secreted form [1], [2]. However, TEM8 is not the only cell surface protein that anthrax toxin uses to enter cells. Capillary morphogenesis gene 2 protein (CMG2), a protein similar to TEM8, was found to be an avid receptor as well [4]. Indeed CMG2 proved a stronger anthrax toxin receptor than TEM8 [5]. CMG2, like TEM8, has multiple splice variants. At least two membrane-bound forms of it, the 488-residue and 489-residue proteins, both strongly support anthrax toxin entry [4], [5]. CMG2 was originally identified by way of its enhanced expression in human umbilical vein endothelial cells, and evidence suggests it is a player in angiogenesis [6].

The only definitively known function of TEM8 is its role as an anthrax toxin receptor, and commensurate with that function it is designated ANTXR1. Likewise, the confirmed role of CMG2 is that of anthrax toxin receptor, and therefore it is designated ANTXR2. Each protein has a von Willebrand Factor A (vWA) domain in its extracellular portion, and within this domain each protein also has a metal ion dependent adhesion site (MIDAS). Both features are important for anthrax toxin protective antigen (PA) binding [4], [5], [7].

Anthrax toxin is secreted by *Bacillus anthracis*, a Gram-positive, encapsulated, spore-forming rod [8], [9]. The toxin comprises three proteins: protective antigen (PA), edema factor (EF), and lethal factor (LF). None of the proteins alone is toxic to cells; the toxic assemblies are PA+EF and PA+LF [8], [9]. EF is a calmodulin-dependent adenylyl cyclase that harms cells by producing excessive amounts of cAMP [10]. LF is a Zn-dependent metalloprotease that targets MAP kinase kinases in cytosol; LF can cleave six out of seven of these kinases [11], [12], [13], [14], [15]. This cleavage is detrimental to cells, and its consequences include apoptosis in at least human endothelial cells and the mouse macrophage line RAW264.7 [16], [17], [18], as well as in human melanoma cells [19]. PA itself has no toxic activity, but is crucial because it delivers EF and LF inside cells by receptor-mediated endocytosis, a process that requires functioning ANTXR1 and ANTXR2 [2], [4], [8], [9]. Upon binding the receptors PA is cleaved by furin, releasing a 23-kDa amino-terminal fragment [20], [21]. The 63-kDa PA remains bound and forms a heptamer, which then binds three molecules of EF, LF, or both [22], [23], [24]. The receptor-toxin complex is then internalized. Upon exposure to acidic pH in an endosome the heptamer changes its conformation, subsequently translocating EF and LF into cytosol [8], [9]. Acidification of the endocytic pathway is indispensable for the intoxication process [25].

The natural ligands for TEM8 and CMG2 have not been precisely identified. It is known, however, that TEM8 interacts with cleaved C5 domain of collagen α3 (VI) [26], while CMG2 can interact with collagen IV and laminin [6]. But the functional significance of these interactions remains obscure. The suggested native role for the two proteins is in neo-angiogenesis, a complex process crucial not only for normal development, but also for many pathological states, such as tumor development and wound healing [9], [6], [27], [28]. Notably, TEM8 shows enhanced expression in endothelia of certain colon carcinomas, suggesting its role in tumor vasculature formation [1], [29]. TEM8 expression has also been studied in some phagocytes [30].

We have carried out extensive variant-specific expression analysis of human TEM8 by nested as well as real-time PCR. We report here that TEM8 transcripts show ubiquitous expression, albeit at different levels. We also report identification of two new transcripts, one of which encodes a fully functional anthrax toxin receptor.

## Materials and Methods

### Reagents and Cells

Human prostate and fetal brain Smart RACE Marathon Ready cDNAs, human multiple tissue cDNA panels, and plasmid pIREShyg3 were purchased from Clontech (Palo Alto, CA). The template in Marathon Ready cDNAs is double-stranded, but that in the tissue panels is first strand synthesized (reverse transcribed) RNA-DNA hybrid. The reagents for PCR (polymerases, dNTPs, and buffers) were from Clontech, Invitrogen (Carlsbad, CA), and Takara (Japan). Primers were made by the FDA Core Facility (Bethesda, Maryland), Sigma-Genosys (Houston, Texas), or Invitrogen (Carlsbad, CA). Restriction enzymes and the ligation kit were from New England Biolabs (Ipswich, MA). Supercompetent XL1-Blue cells were from Stratagene (La Jolla, CA). Functionally receptor-negative cell line PR230 and its parental line, WTB111, have been reported before [3].

### Primers

For TEM8 V1, the primers were based on the reported longest original transcript (NCBI accession number, NM_032208; 5540 bp; coding sequence, 144–1836; 564 aa). For nested PCR, the primary and secondary (nested) PCR primers were designed to partially overlap (Table 1). The primers s21 and as 1842 amplify an 1848-bp V1 fragment containing the coding sequence. For analysis of V2 and V3, the sense primers were the same as for V1, but the antisense primers were specific, each representing the unique 3′ region of V2 (NCBI accession number, NM_053034; 1454 bp; coding sequence, 144–1247; 368 aa), or V3 (NCBI accession number, NM_018153; 2143 bp; coding sequence, 144–1142; 333 aa). All primers were assessed for any secondary structures and self-complementarity using the Oligonucleotide Calculator (http://www.basic.northwestern.edu/biotools/oligocalc.html).

**Table 1:** A directory of primers used to analyze different splice variants of ANTXR1 (TEM8).

Primer	Var	Sequence	PCR
T8V1-S3	1–5	5′TTGCTTCCGGGGAGTTGCGAGGGAGCG	1°
T8V1-S21	1–5	5′GAGGGAGCGAGGGGGAATAAAGGACCC	2°
T8V1-AS1744	1	5′GGTGGAAGGTGGGGACGG	2°
T8V1-AS1738	1	5′GGAAGGTGGGGACGGGATGG	1°
T8V1-AS1728	1	5′TGGGGACGGGATGGGAGGGGTAG	1°
T8V1-AS1716	1	5′GAGGGGTAGGGGCGCTGGGGG	2°
T8V1-S1711	1	5′CCCCGCCCCCCAGCGCCCCTAC	1°
T8V1-S1726	1	5′CCTACCCCTCCCATCCCGTCCC	2°
T8V1-AS3695	1	5′CGCCCGTGGTCCCTGACTAAGGCA	1°
T8V1-AS3670	1	5′GCACTCTGCCTAGATCTATTTTCCCCTG	2°
T8V1-AS1859	1,4,5	5′ACATCTCCTGAAGTTTCTGAGAGAGCC	1°
T8V1-AS1842	1,4,5	5′TGAGAGAGCCCAGAGCAGGAACTTTGG	2°
T8V1-AS1919	1,4,5	5′GTGTGAAGGTCAGTGGGCTTTATCACC	1°
T8V1-AS1903	1,4,5	5′GCTTTATCACCACTCCTCTTCTCTAAC	2°
T8V2-AS1273	2	5′GGGCTGTGTTAGGTTATCTGTTTCTGTGGG	1°
T8V2-AS1251	2	5′TCTGTGGGATTTCTTTCTTTCTTCTTCTTG	2°
T8V3-AS1374	3	5′CCCACCGCATGGAGTGATTATGTAGCC	1°
T8V3-AS1352	3	5′GTAGCCAATAAAGTGCCTCCAGTAAGG	2°

**S,** sense; **AS,** antisense. **1°,** primary PCR. **2°,** secondary (nested) PCR. The 5′ sense primers S3 and S21 are common to all TEM8 variants. The pairs of sense and antisense primers for primary and nested PCR had partial overlaps. V2-as1251 and V2-as1273 are 30 nucleotides long. All other primers are 27 nucleotides long. The expected amplicons after 2° PCR are: V1, 1848 bp (with as1842); V2, 1260 bp; and V3, 1358 bp.

The primers were resuspended in 1 mL of Milli-Q water (Millipore) and incubated at 70°C for 15–20 min to ensure thorough dissolution and inactivation of any accidental contamination with DNase. Appropriate dilutions were made, typically 1∶25, to measure absorbance at 260 nm. The primers were quantified by UV spectrophotometry at 260 nm wavelength. All primers were then diluted to a working concentration of 10 µM.

### Toxins

PA+FP59 was the toxin for cytotoxicity assays. FP59 is a recombinant toxin that carries the PA-binding amino terminal domain of LF and the catalytic domain of *Pseudomonas aeruginosa* exotoxin A. Unlike PA+LF, PA+FP59 can kill all cells. PA was also purchased from List Biological Laboratories (Campbell, CA).

### Nested PCR

The following steps were taken to control for systematic and random experimental variations in PCR results: **1)** For comparison of TEM8 transcript expression levels, all reagents and supplies for PCR were from the same source. **2)** The PCR reaction mixture was always 50 µL, prepared by mixing 5 µL of cDNA template with 45 µL of master mix. The master mix contained (per sample): 1 µL each of 10 µM primers (10 pmol), 5 µL of 10× polymerase buffer, 1 or 4 µL of dNTP stock (2.5 mM or 10 mM each), 1 unit of DNA polymerase (1 µL for Clontech’s Advantage-2 polymerase; 0.25 µL for TaKaRa Taq polymerase), and 33 or 36 µL of PCR grade water. **3)** The same type of 200-µL tubes were used for all PCR reactions. **4)** All PCR cycles were done in the same machine under identical conditions (MyCycler, Bio-Rad, Hercules, CA). **5)** The primary PCR protocol was: 1) 94°C for 25 sec. 2) 72°C for 3 min. 3) 6 times to step 1. 4) 94°C for 25 sec. 5) 72°C for 3 min. 6) 29 times to step 4. 7) 70°C for 5 min. 8) 4°C until removed. **6)** The secondary PCR differed only in the number of cycles: step 3 was 4 times to step 1, and step 6 was 24 times to step 4. For step-wise amplification, the secondary PCR was done in increments of 5 cycles after the first 5. To do so, step 6 repeats were 4, 9, 14, 19, and 24. At the end of each repeat, 4 µL aliquots were removed and the tubes put back for the next 5 cycles. The samples were immediately subjected to agarose gel analysis, or were frozen at –20°C for later analysis.

### Real-time PCR

The QuantiFast SYBR Green PCR Kit was used for qPCR (Qiagen, Gaithersburg, MD). The qPCR machine used was AB7300 (Applied Biosystems/Life Technologies, Carlsbad, CA). The reactions and protocols were performed as directed by the manufacturers. The QuantiFast SYBR green PCR master mix has ROX passive reference dye, which is necessary for Applied Biosystems real-time PCR cyclers. Each reaction mixture contained 12.5 µl of 2X QuantiFast SYBR green PCR master mix, 8.5 µl of RNase-free water, 1 µl each of the sense and antisense primers, and 2 µl of template cDNA. The PCR protocol comprised initial 5 min at 95°C to activate HotStarTaq Plus DNA polymerase, and then 40 cycles of 30-sec denaturation at 95°C and 60-sec annealing/extension at 70°C. Data acquisition was performed during combined annealing/extension step. Melting curve analysis was done immediately after the PCR. The SDS v1.2 software (Applied Biosystems) was used for data analysis. Real-time analysis of β-actin was included as the normalization standard. C_t_ values were determined using the Applied Biosystems software as directed. TEM8 splice variant expressions were compared in terms of ΔC_t_ values, calculated by subtracting the β-actin C_t_ values from the TEM8 variant C_t_ values. Two independent real-time PCRs were done to get the average values.

### Electrophoresis

All analytical and preparative gels were made with Ultrapure agarose (Invitrogen, Carlsbad, CA). The gels were 1% in 1X TAE buffer, and the electrophoresis was also in 1X TAE buffer. For analytical gels, 3 µL of primary or secondary PCR samples were used. For quantitative comparison, analytical gels were made fresh, and all PCR reaction mixtures for fetal and adult tissue panels were loaded on the same gel. Such gels were run for the same length of time. For visualization the gels were stained in water containing 0.2 µg/mL of ethidium bromide solution, made by mixing 3 µL of ethidium bromide (10 mg/mL stock) in 150 mL of deionized water. The gels were stained for 30 min and destained for 45–60 min with two changes of deionized water. The gels were then photographed for permanent records using Chemi-Doc Imager (Bio-Rad, Hercules, CA). To control for exposure and other visualization variations, all photography was performed at constant camera and other settings.

### Purification of PCR Products

Based on results from analytical gels, those mixtures that had unique bands were purified directly using a PCR product purification kit (Qiagen, Germantown, MD). Those mixtures that had two or more PCR bands were subjected to preparative gel electrophoresis. Long-tooth combs were used to make preparative gels. Sample over-loadings were avoided to retain electrophoretic resolution of DNA fragments that had physical proximity. The bands of interest were then cut out of gels and the amplified DNA fragments purified using a gel extraction kit (Qiagen, Germantown, MD). The final elution volume depended on the amount of products, as judged from the analytical gels, and varied from 30 to 100 µL. The purified DNA fragments were analyzed on gels to assess the degree of purity and recovery. When purity was insufficient or recovery low, the PCR reactions were carried out again, sometimes in duplicate or triplicate, to obtain sufficient amounts for purification.

### Sequencing and Analysis

Purified TEM8 amplicons were sent to a commercial facility for sequencing (UT Austin, DNA Core Facility, Austin, TX). Multiple sense and antisense primers were used to get overlapping sequences, and from these the consensus sequences were assembled with Lasergene software (DNA Star, Madison, WI). The sequences were analyzed by BLASTn, BLASTp, and BLAST-2 (NCBI, Bethesda, MD). BLAT (University of California, Santa Cruz) was also used to determine differential splicing of TEM8 variants, as well as precise determination of splicing boundaries. Multiple DNA and protein sequences were aligned using ClustalW2 (http://www.ebi.ac.uk/Tools/msa/clustalw2/).

### Cloning

The fetal brain SmaI-XbaI fragment of TEM8V4 amplicon obtained with primers s21+as1842 was cloned into the StuI-NheI sites of pIREShyg3 by routine recombinant DNA techniques. The final construct was confirmed by sequencing. Ligation of XbaI ends with NheI ends reconstituted the TGA stop codon of TEM8V4. Plasmid-encoded TEM8 V1 and V2 constructs have been reported before [3].

### Transfection

On day 1, PR230 cells were seeded in 10-cm plates in 10 mL of DMEM. On day 2, when cells were about 30% confluent, cells were transfected with plasmid DNA encoding TEM8 V1, V2, or V4 using Cellfectin as directed by the supplier (Invitrogen, Carlsbad, CA). Briefly, 3 µg of plasmid DNA was mixed with 20 µL of Cellfectin. The medium was replaced with 8 mL of fresh DMEM, the transfection mixture added drop by drop, and thoroughly mixed. Following incubation for 6 h, the medium was replaced with 10 mL of fresh DMEM. Two days later the cells were passed 1∶5 in DMEM containing 250 µg/mL of hygromycin B. PR230 alone and transfected with the vector were also subjected to selection. Full selection took nearly two weeks. Cells were kept at 37°C in humidified chambers with 5% CO_2_. The selected cells were stored in liquid nitrogen.

### Cytotoxicity Assays

On day 1, cells were seeded in 96-well plates at a density of 4000 (WTB111) or 5000 (PR230) cells/well in 150 uL of growth medium (DMEM containing 5% FBS, 2 mM glutamine, and 50 µg/mL gentamicin). On day three, various dilutions of PA+FP59 were added in triplicate. FP59 concentration was constant at 50 ng/mL, but PA concentration varied by 5-fold serial dilutions (2000, 400, 80, 16, 3.2, 0.64, and 0.128 ng/mL). On day 5 (48 h), MTT was added (final concentration, 0.5 mg/mL) to assess cell viability. The incubation with MTT was for 45–60 min. The medium was removed and cells lysed in a lysis solution (isopropanol containing 0.1 N HCl and 0.5% SDS; 100 µL/well). Colorimetric readings were taken at 570 nm, with 650 nm as the reference, in Synergy 2 plate reader (Biotek, Winooski, VT).

### PA Binding and Internalization Assays

WTB111, PR230 alone, and PR230 expressing V1, V2, or V4 cells were seeded in 24-well plates at the density of 150,000/well in 1 mL of DMEM. The next day regular growth medium was replaced with 1 mL of binding medium containing 1 µg/mL of PA. The binding medium was DMEM without bicarbonate but supplemented with L-glutamine (2 mM), penicillin (50 u/mL), streptomycin (50 µg/mL), and BSA (0.5 mg/mL). Incubation with the toxin was at 4°C for 2 h. The toxin medium was then aspirated, cells washed five times with binding medium, and lysed in 150 µL of RIPA buffer (Thermo Scientific, Rockford, IL). The buffer composition was 25 mM Tris-Cl, 150 mM NaCl, 1% NP40, 1% sodium deoxycholate, 0.1% SDS, pH 7.6, and it was supplemented with a commercially available cocktail of protease inhibitors (Roche Applied Science, Indianapolis, IN). The lysates were shaken for 5 min, centrifuged for 20 min (16,000×g, 4°C), and the supernatants recovered for further analysis.

Binding at 37°C was performed identically, except that the binding medium was prewarmed to 37°C before adding PA (1 µg/mL). The incubation with the toxin was for 1 h. The cells were then placed on ice, toxin medium removed, washed, and lysed as described above.

### Western Blotting

Total protein in cell lysates was determined using a commercially available Bradford assay kit (Bio-Rad, Hercules, CA). Lysates from the 4°C PA binding assays were subjected to 7.5% SDS-PAGE to resolve PA83 and PA63. The lysates from the 37°C binding assays were subjected to SDS-PAGE in a 4–15% gradient gel to resolve PA83, PA63, and the PA63 heptamer. Equal amounts of total protein (40 µg/mL) were used for SDS-PAGE. The proteins were then transferred to nitrocellulose filters. The filters were incubated at room temperature for 1 h in blocking solution (5% nonfat dry milk in PBS-Tween20) at room temperature, the solution removed, and the filters washed 3 times with PBS-Tween20. Next the filters were incubated for 3 h at room temperature with anti-PA antibody (rabbit polyclonal, 1∶5,000 dilution in PBS-Tween20). The antibody was removed, filters washed 3 times, and incubated with HRP-conjugated goat anti-rabbit antibody (1∶10,000 dilution in PBS-Tween20). Following removal of secondary antibody, the filters were washed again, processed using an ECL Western blotting kit, and exposed to X-ray films (CL-Xposure) for image development (Thermo Scientific, Indianapolis, IN).

## Results

### Identification of a New Membrane-bound Variant of ANTXR1 (TEM8)

The reported original TEM8 (ANXR1) splice variants encode proteins of 564, 368, and 333 residues, respectively designated V1, V2, and V3 [2], [5]. V1 and V2 are single-pass integral membrane proteins and V3 secreted. Both V1 and V2 function as anthrax toxin receptors, while V3 does not [2], [3]. TEM8 was originally discovered as one of nine tumor endothelial markers that showed enhanced expression in colon tumor endothelia [1]. Later findings showed that TEM8 mRNA also shows elevated expression in certain other tumor endothelia [29]. However, a detailed expression analysis of each of the three transcripts has been lacking. For this work we analyzed the expression of each variant in a large number of human tissues using commercially available cDNA panels. Using variant-specific primers, we analyzed the presence of full-length TEM8 transcripts, which required nested PCR.

**Figure 1:**
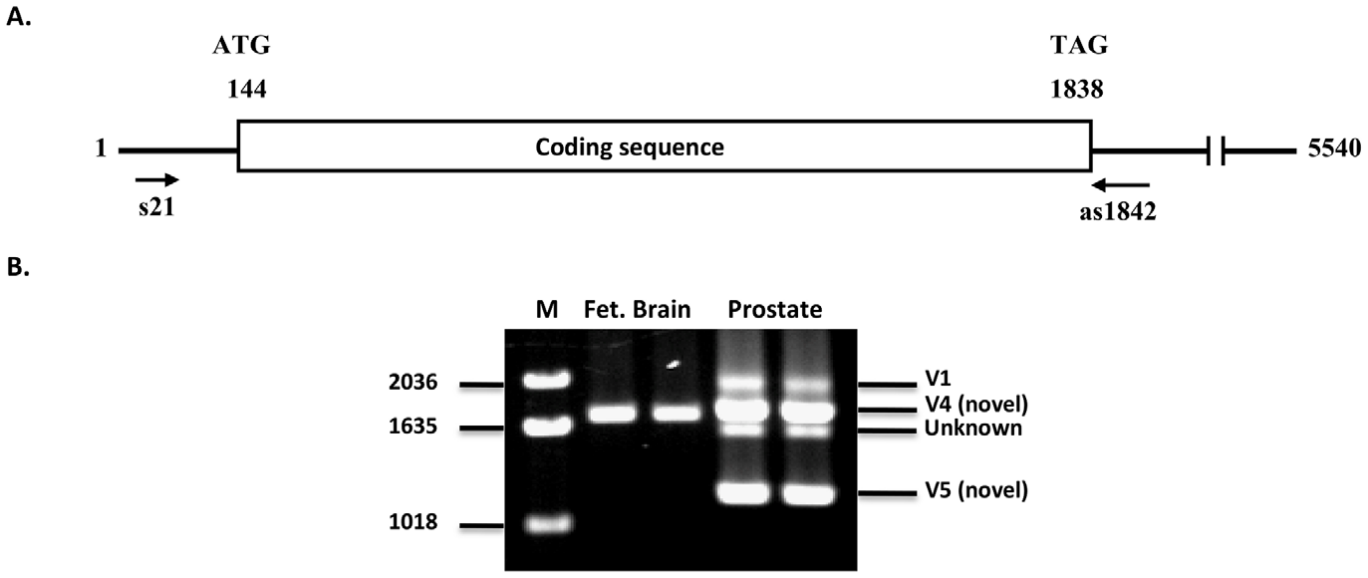
Amplification of ANTXR1/TEM8 from prostate and fetal brain cDNA with V1-specific primers. A ) Schematic representation of V1 transcript features. The positions of nested PCR primers s21 and as1842 are shown. Numbering is based on the original sequence reported by St. Croix et al. (40). **B**) Analysis of amplicons following nested PCR. The expected V1 amplicon was 1848 bp. But the primers amplified a unique 1740 bp new splice variant, which we have designated V4. Prostate had V4 as well as a new variant, V5. Repeated attempts to sequence the unknown fragment failed. The PCR was carried out in duplicate for each template. **M,** molecular weight markers.

For V1 transcript analysis, the primary PCR primers were s3+as1859 (Table 1, Figure 1A). We have based all primer and other numbering on the original 5540-nucleotide transcript for V1 (accession number, NM_032208; ORF, nucleotides 144-1835), originally reported when the gene was first discovered [1]. Initially we ran a pilot analysis using human fetal brain and prostate Marathon Ready cDNA. But PCR with these primers resulted in no amplification. We think this was partly because of low concentration of template cDNA, but could not rule out the possibility that the V1 transcript may be a rare one. We therefore opted to do nested PCR with primers s21+as1842 (Table 1, Figure 1A). This resulted in definite amplification of a unique fragment from the fetal brain cDNA (Figure 1B). The unique amplicon from fetal brain appeared smaller than the expected 1848-bp fragment (nucleotides 21–1868). Indeed sequencing revealed this fragment to be 1740 bp, 108 bp shorter than the expected V1 amplicon. We designated it TEM8 variant 4 (V4).

**Figure 2:**
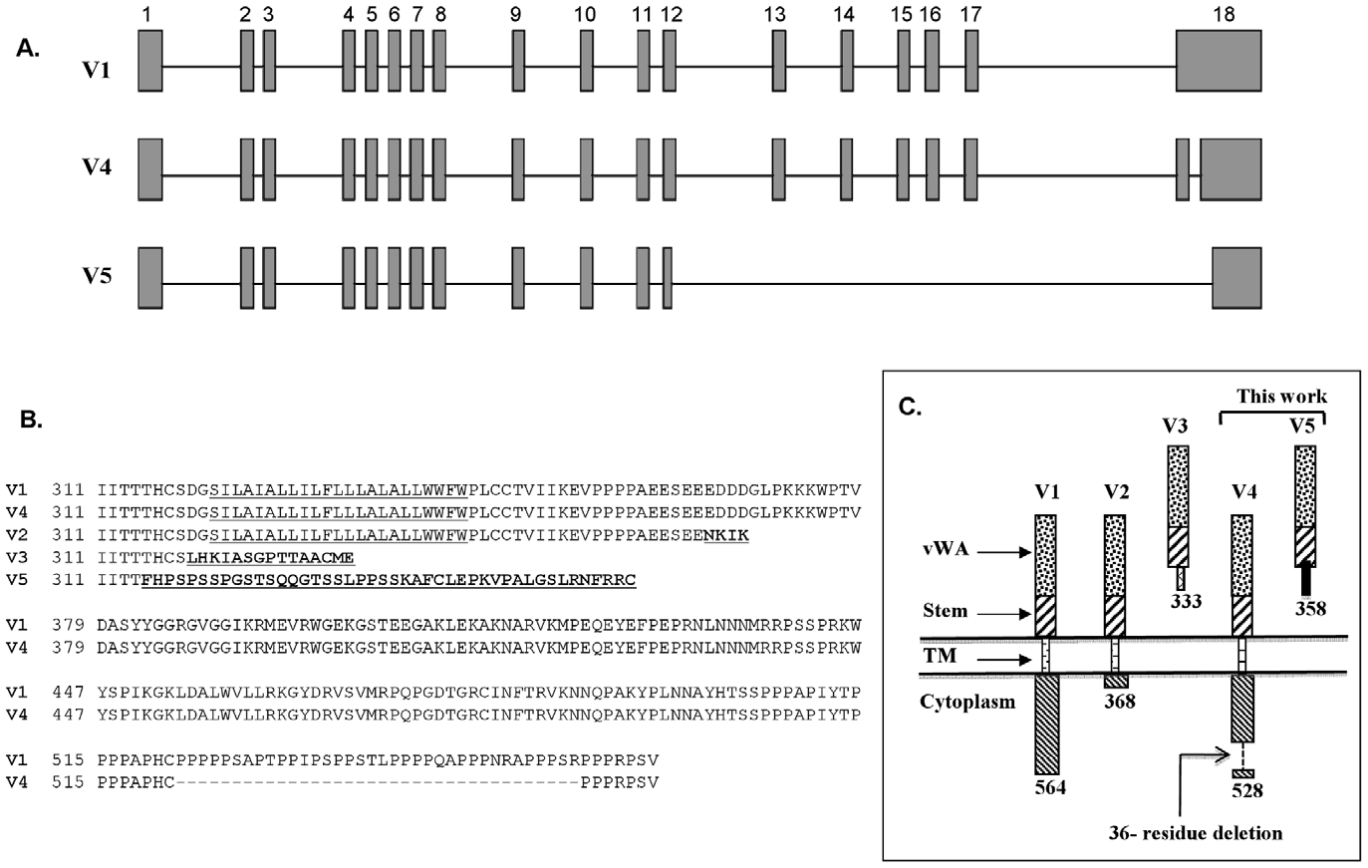
Comparison of ANTXR1/TEM8 variantsV1, V4 and V5 differential splicing. **A**) Differential splicing of new variants V4 and V5 relative to V1. The gene comprises 18 exons with respect to V1 mRNA. V4 results from split splicing within exon 18, excluding a 108-bp segment, but it has the same stop codon as V1. V5 differential splicing occurs by partial skipping of exon 12 and 18 and complete skipping of exons 13–17. This results in a frame shift and consequently V5 acquires a new downstream stop codon. **B**) Sequence comparison of TEM8 receptor variants. The transmembrane helix (TM)) is underlined. The sequence differences are in underlined bold. V4 differs from V1 only in that it has a 36-residue in-frame deletion. V5, like V3, has no TM, and is therefore likely secreted. **C**) A schematic representation of membrane-bound and secreted variants of ANTXR1/TEM8. Same patterns signify identity of sequences and different ones divergence. The putative secreted variants V3 and V5 do not have any alternative membrane-spanning helices in their unique carboxyl-terminal segments.

We analyzed the V4 sequence by BLAST, BLAST-2, and BLAT to precisely determine its splicing boundaries. This analysis revealed that differential splicing of V4 relative to the reported V1 transcript is within exon 18, the last one. The intron 17/exon 18 boundary is the same for V1 and V4. Apparently V4 differential splicing comes about by recruiting new splice donor and acceptor sites within exon 18, resulting in exclusion of the 108-bp segment from the coding sequence of V1. This alternative splicing retains the V1 reading frame, and therefore V4 differs from V1 only in that it has an internal 36-residue in-frame deletion spanning residues 522–557 (Figure 2A, B & C). Thus, V4 is a new 528-residue membrane bound form of TEM8/ANTXR1 (GenBank, JX424838).

PCR with the same primers amplified multiple fragments from the prostate template (Figure 1B), including one identical to the unique fetal brain amplicon, V4. Sequencing confirmed that the prostate fragment corresponding to V4 is indeed the same; that is, it lacks the same 108-bp segment from the coding region. We also isolated the amplicon immediately above the V4 band (labeled V1 in Figure 1B), sequenced it, and found that it indeed is V1; it had the 108-bp segment missing in V4.

### Identification of TEM8 V5, a New Secreted Variant

As already stated, nested PCR analysis of pooled prostate cDNA with the primer sets s3+as1859 and s21+as1842 resulted in multiple amplicons, one of them identical to the 1740-bp fetal brain fragment. However, prostate had a unique smaller amplicon of about 1300 bp. Sequencing revealed this amplicon to be 1361-bp long. Analysis of this sequence by BLAST, BLAST-2, and BLAT revealed that the amplicon represents a new splice form of TEM8, with the same start codon as the other variants. However, differential splicing of this variant comes about by recruiting an alternative splice donor site within exon 12, ten nucleotides upstream of the one for V1. This site then splices with a new acceptor site within exon 18, as shown in Figure 2A. The overall consequence of this is that exons 13–17 are altogether skipped and the reading frame is shifted. The ORF in this sequence is 358 residues, which diverges from the other forms after residue 314, right before the transmembrane helix (Figure 2B). We have designated this TEM8 splice variant V5 (GenBank, JX424839). Like V3, V5 does not have the tramsmembrane helix that V1, V2, and V4 do. It also does not have any other predicted transmembrane helix. We therefore conclude that V5 is a secreted form of TEM8. The divergent carboxyl-terminal segments of V3 and V5 are unique, but otherwise they are identical to the membrane-bound TEM8 proteins. Figure 2C schematically shows the relative topological positioning of the three previously reported variants of TEM8, as well as the two new ones reported in this work.

Prostate also had an amplicon slightly below the 1740-bp one (Figure 1B). But we have not established its identity; repeated attempts at isolating and sequencing it failed. Nonetheless, we cannot rule out that it may be yet another variant of TEM8.

### Analysis of Full-length TEM8 V1, V4, and V5 by Nested PCR

Nested PCR results with human fetal brain and prostate revealed a highly differential profile of TEM8 expression in these two developmentally and histologically distinct tissues. To ascertain a more precise expression profile of each TEM8 transcript, we analyzed a large number of normal human tissues. To do that we used one normalized cDNA set representing 12 digestive system tissues. A second set comprised cDNAs from 8 fetal and 8 adult tissues, 6 of them cognate pairs. For this analysis we used the same V1-specific primers that revealed V4 and V5.

**Figure 3:**
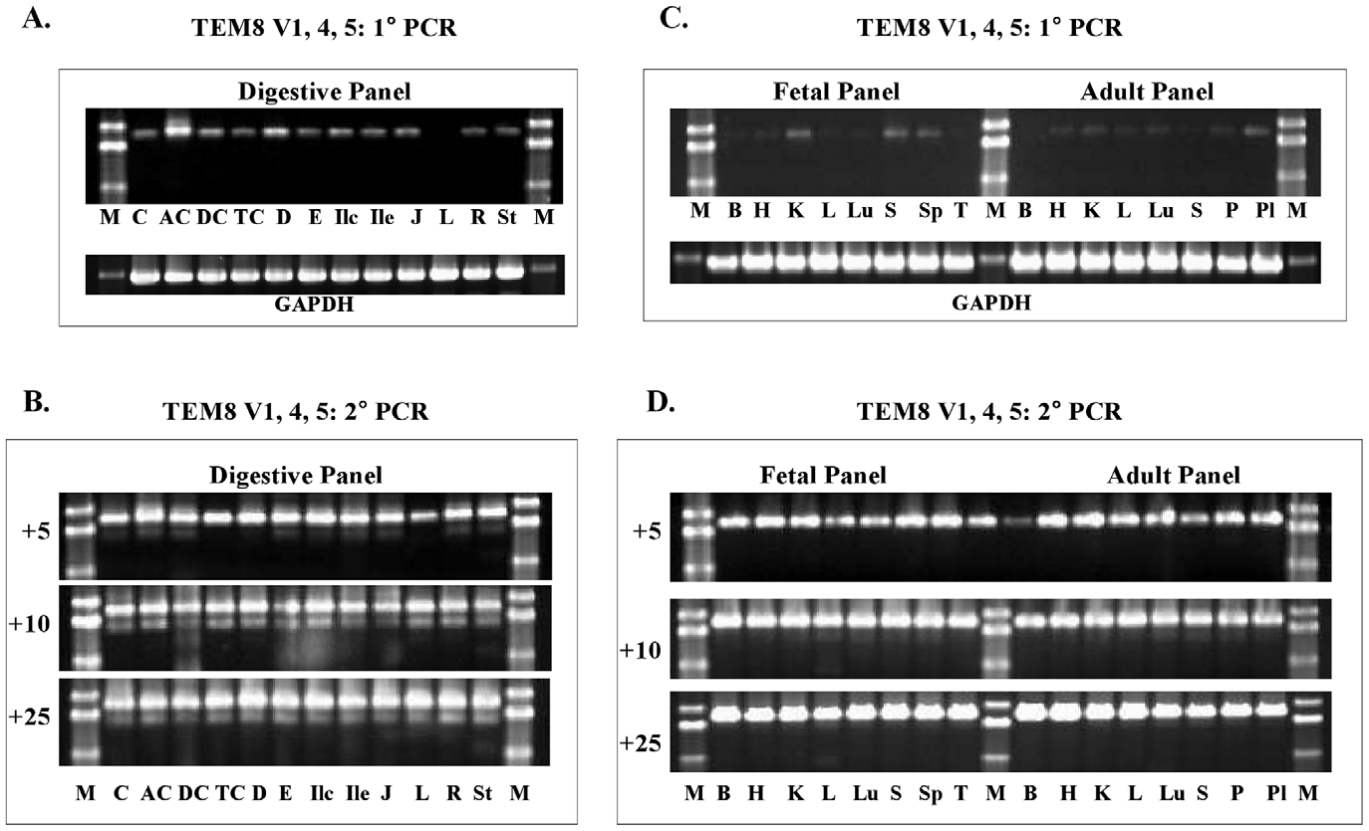
Analysis of TEM8 V1, V4, and V5 expression in human tissue panels. **A & B,** Digestive tissue cDNA panel. **C & D,** Fetal and Adult tissue cDNA panels. The primer pairs used were as for the prostate and fetal brain analysis (Figure 1). There was little or no amplification following 1°PCR, but 2° PCR resulted in definite bands. The number of 2° PCR cycles is shown on the left of each set. The results show that only V4 amplified; V1 and V5 transcripts were absent. **Digestive tissue panel:** **C,** cecum; **AC,** ascending colon; **DC,** descending colon; **TC,** transverse colon; **D,** duodenum; **E,** esophagus; **Ilc,** ileocecum; **Ile,** ileum; **J,** jejunum; **L,** liver; **R,** rectum; **St,** stomach. **Fetal and adult tissue panel: B,** brain; **H,** heart; **K,** kidney; **L,** liver; **Lu,** lung; **P,** pancreas; **Pl,** placenta; **S,** skeletal muscle; **Sp,** spleen; **T,** thymus.

The most prominent band following nested PCR was confirmed to be V4, not V1. As shown in Figure 3, V4 showed ubiquitous expression in digestive, as well as the fetal and adult tissues tested. Upon primary PCR, there appeared noticeable differences in the variant’s expression levels. However, this was not a quantitative analysis, and therefore we could not rely on the differences in expression by this approach. To more precisely determine the expression levels we did real-time PCR.

### Specific Analysis of Full-length TEM8 V1 Transcript by Nested PCR

As described above, nested PCR primers specifically meant to amplify the 1848-bp V1 fragment, which carries the complete coding sequence for this variant, failed to do so, except in prostate. Instead they revealed a new ubiquitous transcript that encodes a 528-residue membrane-bound form of the protein, as well as a putative secreted form unique to prostate (Figures 1 and 3). This initially led us to conclude that V1 transcript may be rare, and that colon carcinoma endothelial cells from which it was first identified may be among the rare tissues that have it [1].

**Figure 4:**
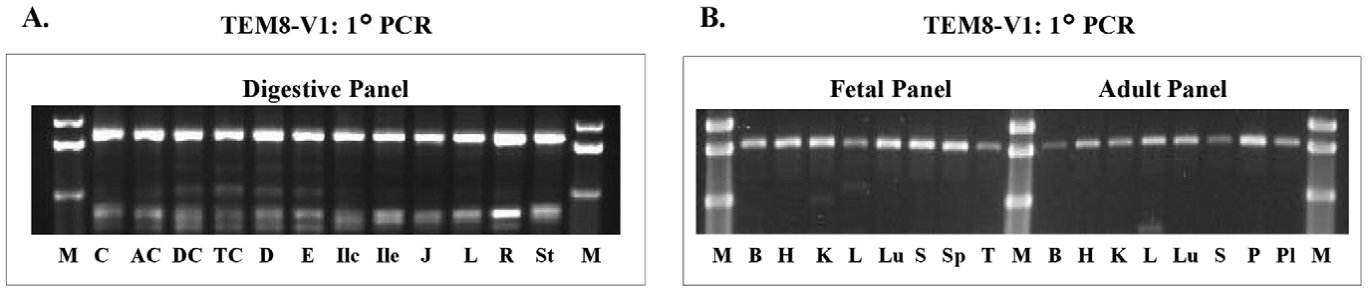
Amplification of V1 with unique V1-specific antisense primers. The sense primer was the same as for primary PCR of other variants, but the antisense primers were specific for the 108-bp segment absent in V4, as well as other variants. Because the amplification was quite robust upon primary PCR, nested PCR was not required. **A, Digestive tissue panel:** **C,** cecum; **AC,** ascending colon; **DC,** descending colon; **TC,** transverse colon; **D,** duodenum; **E,** esophagus; **Ilc,** ileocecum; **Ile,** ileum; **J,** jejunum; **L,** liver; **R,** rectum; **St,** stomach. **B, Fetal and adult tissue panels: B,** brain; **H,** heart; **K,** kidney; **L,** liver; **Lu,** lung; **P,** pancreas; **Pl,** placenta; **S,** skeletal muscle; **Sp,** spleen; **T,** thymus.

Nonetheless, we allowed the possibility that unexpected absence of V1 from so many tissues may be an artifactual anomaly, and therefore decided to further assess its apparent absence. To do so, we made sense and antisense primers specific for the 108-bp region unique to V1, but absent from V4 and V5, as well as V2 and V3. We used these primers in conjunction with s3 and s21, as well as other sense and antisense primers to amplify V1 (Table 1). Indeed these attempts resulted in definite bands of the expected sizes in all tissue cDNA templates (Figure 4). Sequencing confirmed that these fragments carried the 108-bp segment unique to V1. We therefore conclude that the absence of V1 upon first analysis was anomalous. However, we are unable to explain why the antisense primers as1859 and as1842, both specific for the reported V1 transcript, in conjunction with s3 and s21 failed to amplify the expected V1 amplicon in any of the 28 tissues represented in the three cDNA panels.

### Nested PCR Analysis of Full-length TEM8 V2 Expression

**Figure 5:**
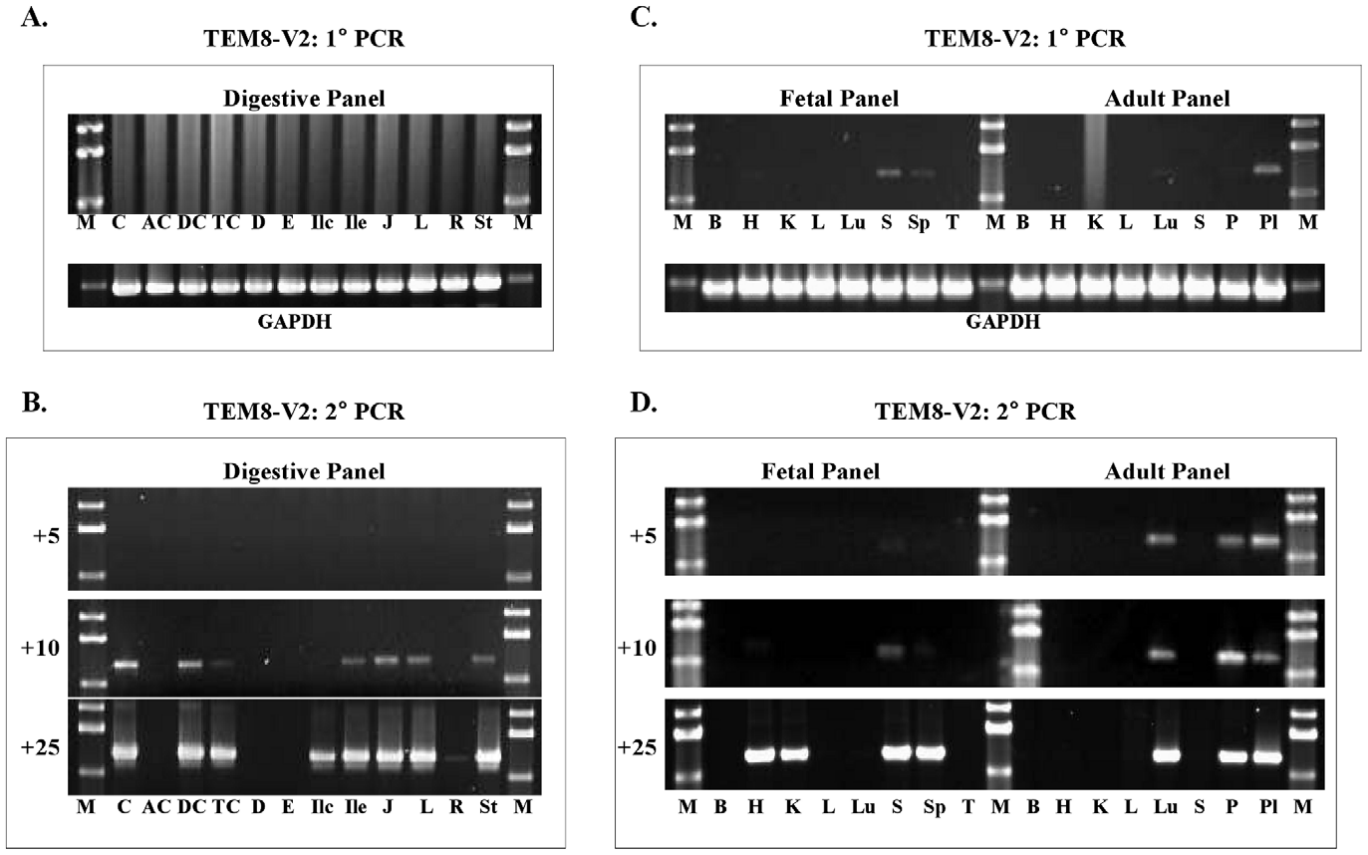
Analysis of TEM8 V2 transcript by stepwise nested PCR. **A & B**, Digestive tissue cDNA panel. **C & D,** Fetal and Adult tissue cDNA panels. The primer pairs used for 1° and 2° PCR were s3+as1273 and s21+as1251, respectively. The number of 2° PCR cycles is shown on the left of each gel picture. **Digestive tissue panel:** **C,** cecum; **AC,** ascending colon; **DC,** descending colon; **TC,** transverse colon; **D,** duodenum; **E,** esophagus; **Ilc,** ileocecum; **Ile,** ileum; **J,** jejunum; **L,** liver; **R,** rectum; **St,** stomach. **Fetal and adult tissue panels: B,** brain; **H,** heart; **K,** kidney; **L,** liver; **Lu,** lung; **P,** pancreas; **Pl,** placenta; **S,** skeletal muscle; **Sp,** spleen; **T,** thymus.

ANTXR1/TEM8 V2 encodes a 368-residue membrane-bound form of the protein, and this is also the form first identified as an anthrax toxin receptor [2]. We analyzed this transcript using specific primers that amplify a 1258-bp segment carrying the entire coding sequence. As shown in Figure 5A and B, this variant appeared to show highly selective expression. However, quantitative analysis by real-time PCR showed that the variant is actually present in all tissues, but its levels differ from tissue to tissue.

### Nested PCR Analysis of Full-length ANTXR1 V3 Expression

**Figure 6:**
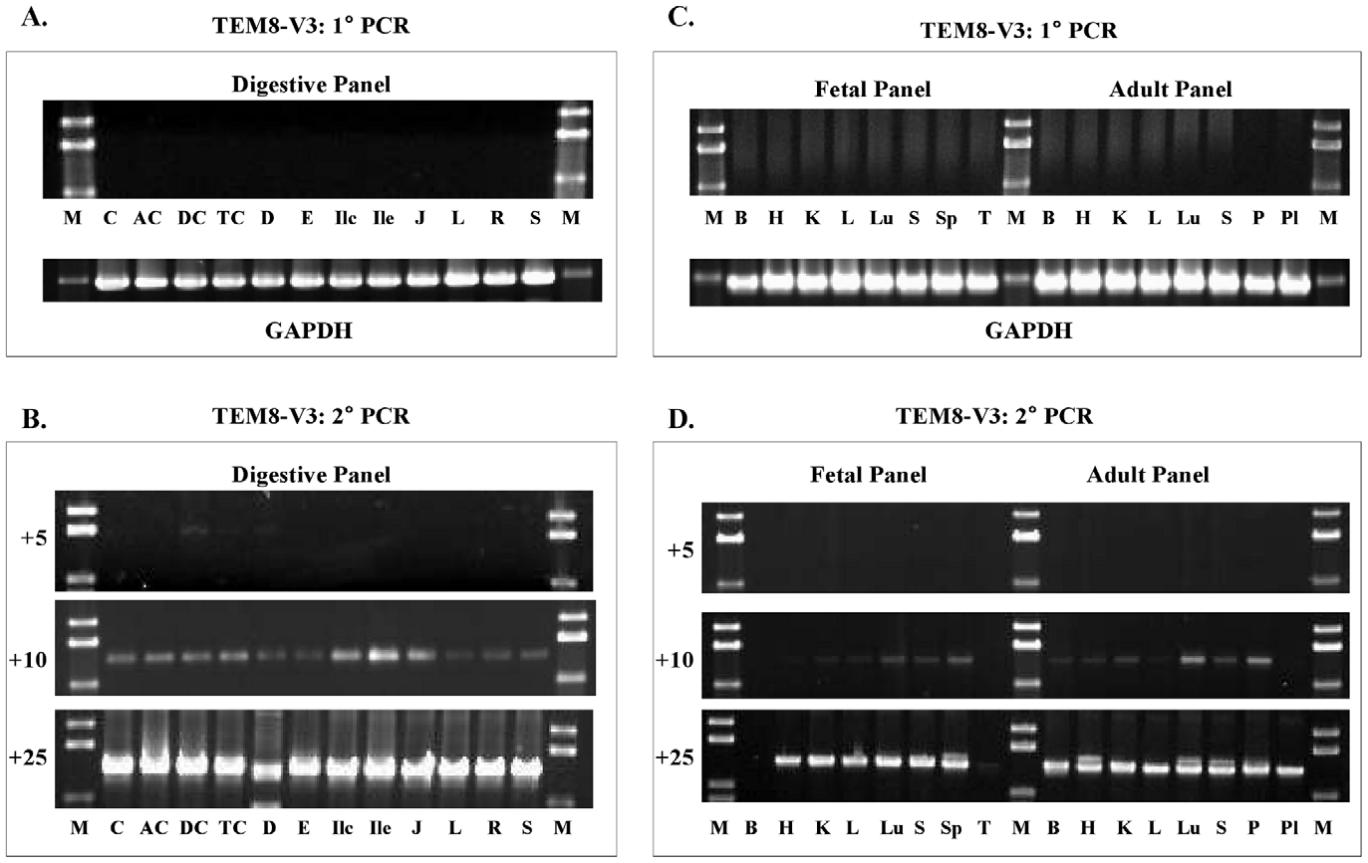
Nested PCR analysis of TEM8 V3 in human tissue panels. A & B, digestive tissue cDNA panel. **C & D,** Fetal and Adult tissue cDNA panel. The primer pairs used for 1° and 2° PCR were s3+as1374 and s21+as1352, respectively. The number of 2° PCR cycles is shown on the left of each gel picture. V3 shows broad expression; all tissues have it, although the amounts vary. **Digestive tissue panel:** **C,** cecum; **AC,** ascending colon; **DC,** descending colon; **TC,** transverse colon; **D,** duodenum; **E,** esophagus; **Ilc,** ileocecum; **Ile,** ileum; **J,** jejunum; **L,** liver; **R,** rectum; **St,** stomach. **Fetal and adult tissue panels: B,** brain; **H,** heart; **K,** kidney; **L,** liver; **Lu,** lung; **P,** pancreas; **Pl,** placenta; **S,** skeletal muscle; **Sp,** spleen; **T,** thymus.

Splice variant 3 of ANTXR1/TEM8 encodes a 333-residue secreted form of the protein [1]. Consistent with that, the variant fails to support anthrax toxin entry when expressed in a receptor negative host [3]. We analyzed expression of this variant using specific primers designed to amplify a 1358-bp cDNA fragment (Table 1). V3, like V4, showed wide expression (Figure 6). All fetal and adult tissues tested had it, as did all digestive tissues.

### Quantitative Analysis of TEM8 Transcripts by Real-time qPCR

**Figure 7:**
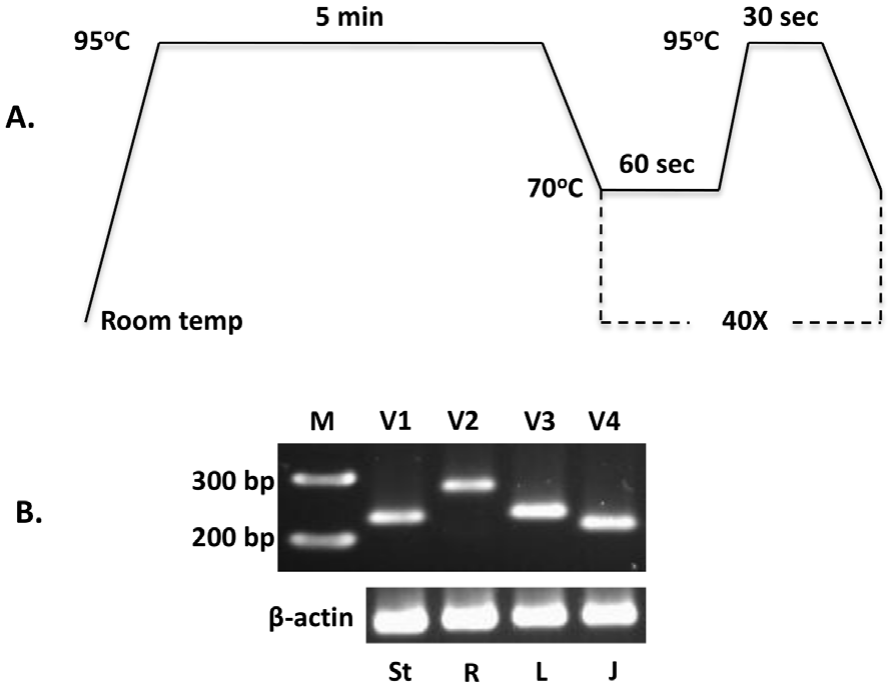
Quantitative analysis of TEM8 splice variants. A, Real-time PCR protocol. **B,** Agarose gel analysis of amplicons. β-actin was used as the normalization standard. The expected amplicons were: V1, 231 bp; V2, 281 bp; V3, 234 bp; V4, 218 bp. Several experiments were done to set the appropriate conditions, and to ensure that the real-time PCR worked satisfactorily and that the expected fragments were amplified. Different tissue cDNAs were used for this purpose. **St,** stomach; **R,** rectum; **L,** liver; **J,** jejunum. We got no reliable results with V5, and therefore excluded it from real-time PCR analysis.

The major goal of nested PCR was to ascertain the status of full-length splice variants of TEM8. Although this analysis revealed some remarkable differences in variant expression from tissue to tissue, notably for V2, the approach is imprecise for quantitative comparisons. To more precisely ascertain the expression levels, therefore, we carried out real-time quantitative PCR as described in Material and Methods. Consistent with real-time qPCR requirements, the target amplicons for this analysis were much smaller –231 bp (V1), 281 bp (V2), 234 bp (V3), and 218 bp (V4) – and the primers used for this work are listed in Table 2. Initially we did several pilot studies to set the proper conditions and to verify the authenticity of target amplicons. Results from one such experiment are shown in Figure 7. As is clear, the target fragments amplified. We then used the same conditions to perform qPCR on the tissue cDNA panels.

**Table 2:** Primers used for variant-specific real-time PCR analysis of TEM8.

Primer	Variant	Sequence
T8V1-S1506	1	5′GATGCCTTGTGGGTCCTACTGAGGA
T8V1-AS1716	1	5′GAGGGGTAGGGGCGCTGGGGG
T8V1-S1021	2	5′CTGCACTCCAGGTCAGCATGAACGATGG
T8V2-AS1273	2	5′GGGCTGTGTTAGGTTATCTGTTTCTGTGGG
T8V3-S1166	3	5′CTCCGGACAGCACACTCCTGAAAAC
T8V3-AS1374	3	5′CCCACCGCATGGAGTGATTATGTAGCC
T8V1-S1506	4	5′GATGCCTTGTGGGTCCTACTGAGGA
T8V4-AS1671	4	5′AGAAGGCCTTGGAGGAGGGCAGTG

**S,** sense. **AS,** antisense. Expected amplicons: V1, 231 bp; V2, 281 bp; V3, 234 bp; V4, 218 bp.

**Figure 8:**
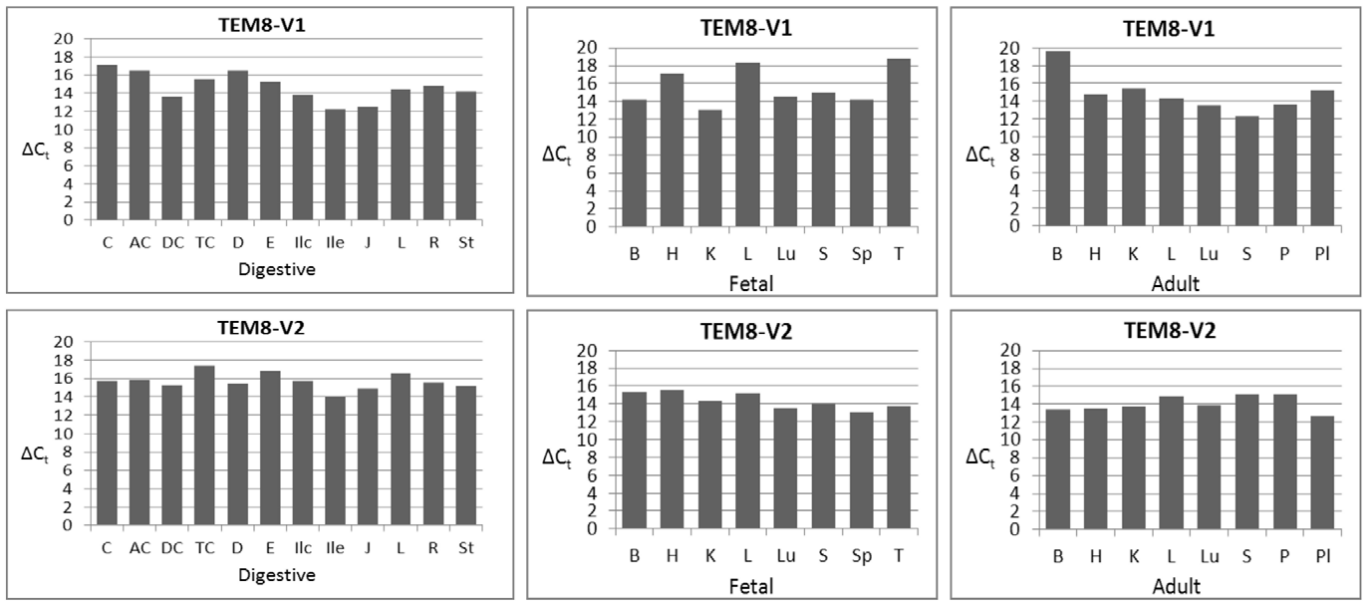
Quantitative analysis of TEM8 splice variants V1 and V2. The analysis was carried out according to the real-time PCR protocol in Figure 7, and as described in Materials and Methods. ΔC_t_ values are the averages of two independent real-time PCRs, calculated by subtracting the C_t_ values for each variant from the C_t_ values for β-actin. **Digestive tissue panel:** **C,** cecum; **AC,** ascending colon; **DC,** descending colon; **TC,** transverse colon; **D,** duodenum; **E,** esophagus; **Ilc,** ileocecum; **Ile,** ileum; **J,** jejunum; **L,** liver; **R,** rectum; **St,** stomach. **Fetal and adult tissue panels: B,** brain; **H,** heart; **K,** kidney; **L,** liver; **Lu,** lung; **P,** pancreas; **Pl,** placenta; **S,** skeletal muscle; **Sp,** spleen; **T,** thymus.

The results in Figure 8 show that V1 is ubiquitous, consistent with the nested PCR results. However, its levels vary from tissue to tissue, at times markedly. Of the digestive tissues, jejunum had the greatest and colon the least amounts of V1, as suggested by the difference of 5 in their ΔC_t_ values. Among the fetal tissues, kidney showed the greatest and thymus and liver the least of V1. Of the adult tissues, skeletal muscle showed much greater expression than brain. There were differences in V1 expression among the cognate pairs of fetal and adult tissues. Fetal brain, for example, had much greater expression of V1 than did adult brain. But there appeared no definitive pattern; some fetal tissues had more of V1 than their cognate adult tissues, and vice versa. Of all 28 tissues analyzed, adult brain had the lowest expression of V1.

Quantitative analysis results for V2 differed from the nested PCR results in that the qPCR revealed presence of V2 in all tissues, whereas nested PCR did not. V2 showed differences in expression from tissue to tissues, but generally these variations were not as remarkable as for V1 (Figure 8).

**Figure 9:**
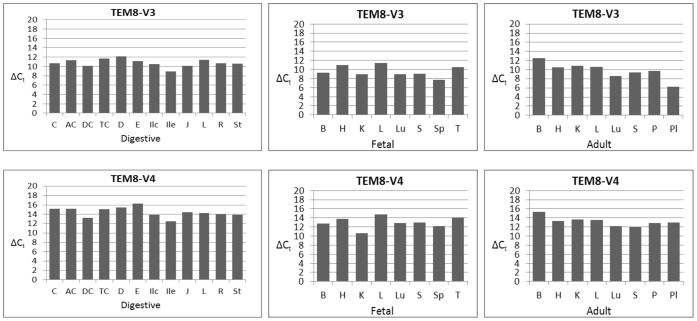
Real-time PCR analysis of TEM8 splice variants V3 and V4. Digestive tissue panel: **C,** cecum; **AC,** ascending colon; **DC,** descending colon; **TC,** transverse colon; **D,** duodenum; **E,** esophagus; **Ilc,** ileocecum; **Ile,** ileum; **J,** jejunum; **L,** liver; **R,** rectum; **St,** stomach. **Fetal and adult tissue panels: B,** brain; **H,** heart; **K,** kidney; **L,** liver; **Lu,** lung; **P,** pancreas; **Pl,** placenta; **S,** skeletal muscle; **Sp,** spleen; **T,** thymus.

Judged by the ΔC_t_ values, V3 generally showed the strongest expression of all variants (Figure 9). Placenta and fetal spleen showed the most robust expression, while adult brain, fetal liver, duodenum, and transverse colon expressed the least. Of the paired fetal and adult tissues, V3 appeared at greater levels in fetal brain than adult brain.

The newly identified splice variant that encodes a membrane-bound form of TEM8, V4, was present in all tissues, consistent with the nested PCR results. Of the digestive tissues, ileum showed significantly stronger expression than did esophagus (ΔC_t_ difference ∼4). Other differences with ileum varied from 1 to 3. Among the fetal and adult tissues, fetal kidney appeared to have markedly greater level of V4 than did adult kidney. The variant also showed stronger expression in fetal brain than did adult brain.

We were unable to amplify the specific V5 fragment by nested or real-time PCR in any of the 12 digestive tissues, 8 fetal tissues, and 8 adult tissues.

### Analysis of V4 Function as an Anthrax Toxin Receptor

**Figure 10:**
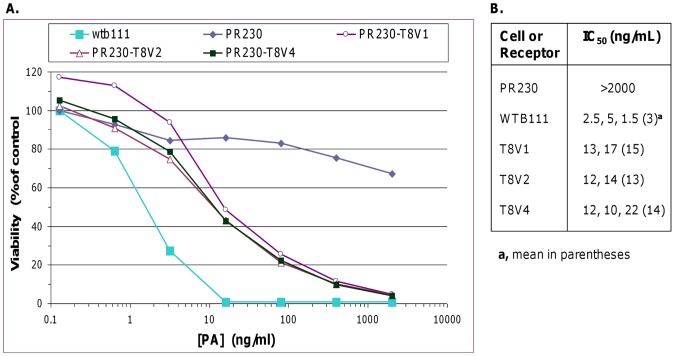
Assessment of TEM8 functionality as an anthrax toxin receptor. A, Sensitivity of cells expressing V1, V2, or V4. The plasmid-encoded receptors were expressed in the receptor-negative host PR230 as described in Materials and Methods, followed by cytotoxicity assays with PA+FP59. **B,** Toxin concentrations required for 50% cell death (IC_50_, ng/mL) for PR230 expressing V1, V2, or V4. The means of 2–3 independent experiments are shown in parentheses. The greater sensitivity of the parental cells, WTB111, is probably due to expression of both receptors, especially CMG2/ANTXR2, which has about 10 times greater affinity for anthrax toxin protective antigen than does TEM8 [40], [4], [33].

The extracellular portion of V4 is identical to those of V1 and V2, both functional anthrax toxin receptors. It has also been shown that TEM8 functions as an anthrax toxin receptor even when its intracellular portion is removed [3]. We therefore reasoned that V4 would also prove a functional toxin receptor, despite the 36-residue in-frame internal deletion in its cytoplasmic domain. To verify, we tested V4 as an anthrax toxin receptor in parallel with the previously reported forms V1 and V2. V4 cDNA was cloned into the mammalian expression vector pIREShyg3 as described in Materials and Methods. V1 and V2 cloning and expression has been described before [3]. The plasmid-encoded receptors were expressed in PR230, a functionally receptor-negative Chinese hamster ovary cell line [3]. As shown in Figure 10, V4 supported anthrax toxin entry as efficiently as V1 and V2, and the toxin IC_50_ for PR230 expressing any of the three receptors was nearly the same. The host cell alone was resistant. WTB111, the parent of PR230, was included as a control for toxicity. A modified form of anthrax toxin, PA+FP59, was used for these assays. FP59 is a recombinant toxin that carries the PA-binding amino-terminal domain of LF and the ADP-ribosyltransferase domain of *Pseudomonas aeruginosa* exotoxin A [31]. The target for exotoxin A is the eukaryotic elongation factor 2, which the toxin inactivates by ADP-ribosylation, thus arresting protein synthesis and killing the cell.

### Assessment of PA Binding, Processing, and Internalization

The overall efficiency of intoxication depends on a series of sequential events that underlie the toxin endocytosis. The notable ones are PA binding to receptors, cleavage by furin to release a 20-kDa amino-terminal fragment, PA63 heptamer formation on the cell surface, heptamer internalization, and routing to an acidic compartment, where the heptamer undergoes conformational changes [9], [22], [25], [32], [33]. It is not inconceivable that various receptor forms could differ in their capacity to support these events, and yet the toxin IC_50_ values may be nearly the same. We therefore assessed PA binding, cleavage by furin, and oligomerization in PR230 cells expressing V1, V2, or V4.

**Figure 11:**
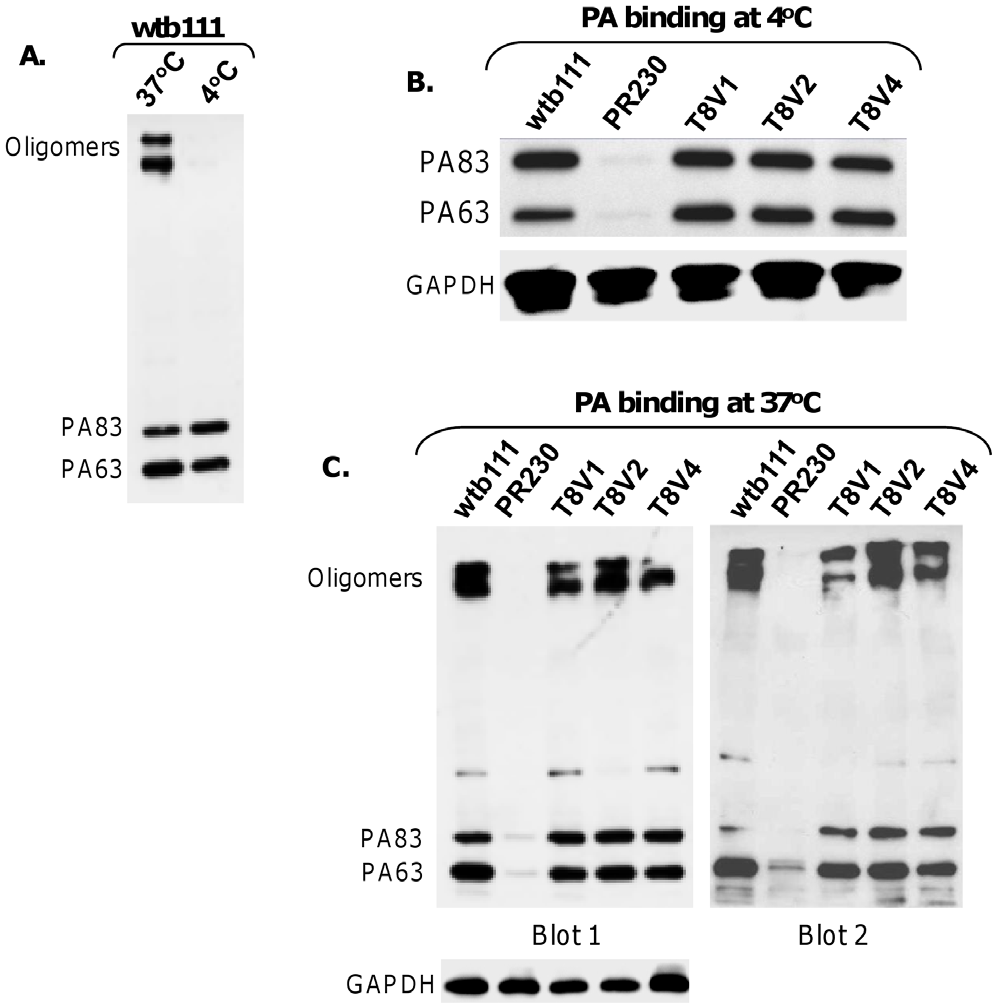
V1, V2, and V4 capacities for anthrax toxin protective antigen (PA, 83 kDa) binding, processing, and internalization. A, PA binding to normal cells (WTB111). At 4°C, PA83 binds the receptors and is cleaved by furin to yield PA63. At 37°C, PA83 not only binds and is cleaved by furin, but enters cells and forms SDS-resistant oligomers. **B,** PA binding and processing at 4°C to PR230 expressing V1, V2, or V4. **C,** PA binding, processing, internalization, and SDS-resistant oligomer formation at 37°C. Two blots of a typical experiment are shown.

The binding assays were done at 4°C and 37°C as described in Materials and Methods. At 4°C membrane trafficking is essentially arrested, and consequently no PA enters cells. But receptor-bound PA83 is still cleaved by furin to form PA63 [3], [32]. The PA63 heptamer that forms on the cell surface resolves into PA63 monomers upon SDS-PAGE. In contrast, at 37°C the heptamer enters cells and becomes SDS-resistant upon exposure to acidic pH in endosomes. Thus, appearance of this heptamer on Western blots can serve as an index of toxin entry. This is shown in a control experiment with WTB111 (Figure 11A). Little, if any, PA63 oligomers appear when the binding experiment is performed at 4°C, but a strong doublet of oligomers appears when the experiment is done at 37°C. With these cells we have consistently observed that two bands representing PA oligomers appear. Such doublets have been reported before [3], [34]. It is also known that aside from the heptamer, PA63 also forms octamers, and such assemblies involve intermediate stages of dimers and tetramers [35]. However, the lower order assemblies do not appear to be SDS-resistant [3], [9], [22], [23], [24], [33].

The 4°C binding assays revealed that V1, V2, and V4 equally support PA binding and its cleavage by furin, as is clear from the PA83 and PA63 bands in Figure 11B. We next assessed the three receptors’ capacity to support entry of PA. To do that we incubated cells with PA at 37°C, followed by processing as for the 4°C assays. As shown in Figure 11C, all three receptors bound PA83 equally well at this temperature as well. Further, the subsequent cleavage of PA83 to yield PA63 was also equally efficient for all three receptors. And the SDS-resistant oligomer formation was also efficient for all three receptors. The oligomers reflect successful entry of the toxin. Overall these results are fully consistent with the finding that all three receptors make PR230 cells sensitive to PA+FP59 to the same extent. Thus, when expressed in PR230, V4 as an anthrax toxin receptor is as efficient as V1 and V2.

## Discussion

Discovered as a gene that showed elevated expression in endothelial cells of colon carcinomas, TEM8 was suggested to be a player in tumor vasculature development [1]. Histological studies that employed *in situ* mRNA hybridization also showed the gene’s enhanced expression in tumor endothelia [29]. However, the relative expression levels of the three reported TEM8 transcripts remained undetermined. Therefore it was unclear whether all three were elevated in tumor endothelia or only one of them. Indeed at that time it was unclear whether TEM8 is expressed in normal tissues. Moreover, *in situ* mRNA hybridizations are not nearly as sensitive as PCR, and thus often fail to detect transcripts expressed at low levels. Variant-specific expression profile of the gene remained undetermined altogether. Once determined as an anthrax toxin receptor [2], it was clear that the gene shows wide expression, given that the toxin can bind and enter a wide variety of cell types [8], [36]. It has also been reported that TEM8 shows differential expression in mouse tissues [37].

The major focus of this work was to ascertain an expression profile of all splice variant mRNAs of TEM8 in human tissues. The analysis employed variant-specific primers and nested PCR. To further ensure this analysis is indeed of those TEM8 transcripts that encode the three reported variants, we used primers designed to amplify fragments that carry the complete coding regions. Our findings show that all tissues analyzed have the previously reported variants, V1, V2, and V3. Although nested PCR showed selective expression of V2, the real-time PCR showed that the variant is indeed expressed in all tissues analyzed. We therefore conclude that V2 is not selectively expressed, and its absence in some cases was due to failure of nested PCR.

Analysis of V1 transcript initially proved problematic; two separate sets of primers specific for the reported V1 sequence failed to amplify it. We are unable to explain this anomaly, but the same primers amplified two novel splice variants of TEM8. One of these, V4, encodes a 528-residue membrane-bound form of TEM8, and the other, V5, a 358-residue protein (Figures 1, 2 & 3). The V5 protein sequence diverges after residue 314, and it does not have the TEM8 hydrophobic transmembrane helix, nor any other predicted one. We therefore conclude that the putative V5 protein is a secreted form of TEM8. Prostate cDNA pools analyzed were the only ones that had V5; neither nested PCR, nor real-time PCR amplified the target fragments of V5 from any of the other 28 tissues analyzed. We therefore conclude that V5 is likely an exceedingly rare splice form of TEM8.

The newly discovered variant V4 shows broad expression, as evidenced by both nested and real-time PCR (Figures 3 & 9). The V4 sequence differs from V1 in that it lacks a 108 bp cDNA segment, resulting in an in-frame deletion of 36-residues (residues 522–557) in the cytoplasmic domain. We therefore exploited this difference to analyze V1 transcript using primers specific for the 108-bp V1 segment. This approach worked; separate sets of sense and antisense primers specific for this region gave the expected amplicons (Figures 4 & 8).

V1 and V2 are proven anthrax toxin receptors [3], [7]. We have shown that V4 too is a functional anthrax toxin receptor. The receptor confers the same degree of sensitivity on PR230 as V1 and V2 (Figure 10). PR230 is a functionally receptor-negative cell line that has been described before [3]. Consistent with these results, anthrax toxin protective antigen efficiently binds V4, is cleaved by furin, is internalized, and forms the SDS-resistant oligomers (Figure 11B, C).

Our results demonstrate that TEM8 expression is not restricted to any particular type of tissue. However, whether the normal and tumor endothelial cells show different expression patterns for all five variants remains to be seen. Another point to note is that we used pooled tissue cDNA panels representing different individuals. Thus, it is impossible to tell whether the gene exhibits individual-specific variations. As an example, we found V5 in one pooled prostate cDNA panel, but not in another (not shown). This suggests the possibility that the gene’s expression profile may even vary from individual to individual. A statistically significant number of individual samples would be required to discern such differences, if any.

An intriguing expression pattern of TEM8 in neuroblastoma cell lines has been reported. In these cells the gene is found as fusion transcripts with various segments of novel neuroblastoma gene 1 (NNG1) [38], [39]. Oberthuer et al. identified 4 such transcripts, each representing the first 14 exons of TEM8 and various segments of NNG1. Revealed by sequencing, the transcripts encode fusion proteins of 388, 411, 412, and 420 residues, all comprising the first 363 residues of TEM8. However, the functional significance, if any, of these fusion transcripts is unclear.

The putative role of ANTXR1/TEM8 in neo-angiogenesis remains dubious. But in mice the gene is dispensable, as evidenced by normal development of TEM8^−/−^ knockout mice [40]. Indeed, the other anthrax toxin receptor, ANTXR2/CMG2, is also dispensable for mouse development, as shown by studies with CMG2^−/−^ mice [40]. Note that CMG2 has also been reported a player in angiogenesis [6], [41]. However, these findings do not necessarily rule out angiogenic role for TEM8 and CMG2 during mouse development. If the two genes are indeed players during mouse developmental angiogenesis, then there exist other players that compensate for the loss of TEM8 and CMG2. In as much as ANTXR1/TEM8 shows enhanced expression in at least some tumor endothelial cells [1], [29], the gene does appear to be a player in tumor vasculature development. Evidence also suggests that TEM8 promotes endothelial cell adhesion and migration, which it seems to achieve by interacting with cell matrix proteins [42].

Many recent findings have significantly unraveled the endocytic pathway of TEM8 and the protein’s interactions with other factors. Wei et al. discovered that LRP6, a single-pass integral membrane protein related to the LDL receptor, interacts with TEM8, as well as the other PA receptor, CMG2 [43]. Anthrax toxin endocytosis is largely via the clathrin-mediated pathway, evidently for both TEM8 and CMG2 [3], [32], [34]. Both receptors cluster in lipid rafts, and TEM8 is palmitoylated and ubiquitinated, modifications that evidently trigger its endocytosis [44]. TEM8 V1 interacts with actin, and this interaction affords more PA binding to this form of ANTXR1/TEM8, although it does not necessarily afford greater sensitivity of cells to anthrax toxin [34], [45]. Some other classical players in endocytosis, such as Cbl and β-arrestins 1 & 2, are also important for PA heptamer formation. In contrast, Grb2 and Dab2 appear unimportant [34]. Surprisingly, AP2, the predominant adaptor for clathrin-mediated vesicle budding at the plasma membrane, seems less relevant to PA entry than AP1, which largely functions in vesicle budding from endosomes and trans-Golgi network [34].

At the molecular and cellular level, the native functions of ANTXR1/TEM8 remain obscure. The relative roles of the receptor’s various isoforms are even more elusive. Is it possible, for example, that relative enhancement or attenuation of TEM8 variants defines the gene’s role in tumor angiogenesis. Is the gene important in vasculature formation during other disease states, such as wound healing and other forms of tissue repair? Liu et al. have shown that TEM8 and CMG2 are not needed for mouse developmental angiogenesis [40]. However, whether these findings can be extended to human development is unknown. They further reported that CMG2 is the major anthrax toxin receptor and that toxin binding to it is 10 times stronger than to TEM8. Evidence also suggests that TEM8 exists in different forms on the cell surface, and that these forms are influenced by actin and the actin binding protein transgelin [46]. Clearly much more research is needed to precisely elucidate the role of TEM8 and CMG2 at the molecular level in disease state and normal vasculature formation.

## References

[1] St Croix B, Rago C, Velculescu V, Traverso G, Romans KE, et al (2000) Genes expressed in human tumor endothelium. Science 289: 1197–1202. 10947988 10.1126/science.289.5482.1197

[2] Bradley KA, Mogridge J, Mourez M, Collier RJ, Young JA (2001) Identification of the cellular receptor for anthrax toxin. Nature 414: 225–229. 11700562 10.1038/n35101999

[3] Liu S, Leppla SH (2003) Cell surface tumor endothelium marker 8 cytoplasmic tail-independent anthrax toxin binding, proteolytic processing, oligomer formation, and internalizations. J Biol Chem 278: 5227–5234. 12468536 10.1074/jbc.M210321200

[4] Scobie HM, Rainey GJ, Bradley KA, Young JA (2003) Human capillary morphogenesis protein 2 functions as an anthrax toxin receptor. Proc Natl Acad Sci USA, 100 (9); 5170–5174. 12700348 10.1073/pnas.0431098100 PMC154317

[5] Scobie HM, Young JA (2005) Interactions between anthrax toxin receptors and protective antigen. Curr Opin Microbiol 8(1): 106–112. 15694864 10.1016/j.mib.2004.12.005

[6] Bell SE, Mavila A, Salazar R, Bayless KJ, Kanagala S, et al (2001) Differential gene expression during capillary morphogenesis in 3D collagen matrices: regulated expression of genes involved in basement membrane matrix assembly, cell cycle progression, cellular differentiation, and G-protein signaling. J Cell Sci 114: 2755–2773. 11683410 10.1242/jcs.114.15.2755

[7] Bradley KA, Mogridge J, Jonah G, Rainey A, Batty S, Young JAT (2003) Binding of anthrax to its receptor is similar to α-integrin-ligand interactions. J Biol Chem 278: 49342–49347. 14507921 10.1074/jbc.M307900200

[8] Leppla SH (2000) Anthrax toxin. In: Aktories K, Just I, editors. Handbook of Experimental Pharmacology: Bacterial Protein Toxins. New York: Springer-Verlag, publisher. 445–472.

[9] Van der Goot G, Young JA (2009) Receptors of anthrax toxin and cell entry. Mol Aspects Med 30 (6): 406–412. 10.1016/j.mam.2009.08.007 PMC2783407 19732789

[10] Leppla SH (1982) Anthrax toxin edema factor: a bacterial adenylate cyclase that increases cyclic AMP concentrations of eukaryotic cells. Proc Natl Acad Sci USA 79: 3162–3166. 6285339 10.1073/pnas.79.10.3162 PMC346374

[11] Duesbery NS, Webb CP, Leppla SH, Gordon VM, Klimpel KR, et al (1998) Proteolytic inactivation of MAP-kinase kinase by anthrax lethal factor. Science 280: 734–737. 9563949 10.1126/science.280.5364.734

[12] Klimpel KR, Arora N, Leppla SH (1994) Anthrax toxin lethal factor contains a zinc metalloprotease consensus sequence which is required for lethal toxin activity. Mol Microbiol 13: 1093–1100. 7854123 10.1111/j.1365-2958.1994.tb00500.x

[13] Pellizzari R, Guidi-Rontani C, Vitale G, Mock M, Montecucco C (1999) Anthrax lethal factor cleaves MKK3 in Macrophages and inhibits the LPS/IFN-γ-induced release of NO and TNF-α. FEBS Lett 462: 199–204. 10580119 10.1016/s0014-5793(99)01502-1

[14] Vitale G, Bernardi L, Napolitani G, Mock M, Montecucco C (2000) Susceptibility of mitogen-activated protein kinase kinase family members to proteolysis by anthrax lethal factor. Biochem J 352: 739–745. 11104681 PMC1221512

[15] Vitale G, Pellizzari R, Recchi C, Napolitani G, Mock M, et al (1998) Anthrax lethal factor cleaves the N-terminus of MAPKKs and induces tyrosine/threonine phosphorylation of MAPKs in cultured macrophages. Biochem Biophys Res Commun 248: 706–711. 9703991 10.1006/bbrc.1998.9040

[16] Kirby JE (2004) Anthrax lethal toxin induces human endothelial cell apoptosis. Infect Immun 72: 430–439. 14688124 10.1128/IAI.72.1.430-439.2004 PMC343952

[17] Park JM, Greten FR, Li ZW, Karin M (2002) Macrophage apoptosis by anthrax lethal factor through p38 MAP kinase inhibition. Science 297: 2048–2051. 12202685 10.1126/science.1073163

[18] Popov SG, Villasmil R, Bernardi J, Grene E, Cardwell J, et al (2002) Lethal toxin of *Bacillus anthracis* causes apoptosis of macrophages. Biochem Biophys Res Commun 293: 349–355. 12054607 10.1016/S0006-291X(02)00227-9

[19] Koo HM, VanBrocklin M, McWilliams MJ, Leppla SH, Duesbery NS, et al (2002) Apoptosis and melanogenesis in human melanoma cells induced by anthrax lethal factor inactivation of mitogen-activated protein kinase kinase. Proc Natl Acad Sci USA 99 (5): 3052–3057. 10.1073/pnas.052707699 PMC122471 11867750

[20] Gordon VM, Klimpel KR, Arora N, Henderson MA, Leppla SH (1995) Proteolytic activation of bacterial toxins by eukaryotic cells is performed by furin and by additional cellular proteases. Infect Immun 63: 82–87. 7806387 10.1128/iai.63.1.82-87.1995 PMC172960

[21] Klimpel KR, Molloy SS, Thomas G, Leppla SH (1992) Anthrax toxin protective antigen is activated by a cell-surface protease with the sequence specificity and catalytic properties of furin. Proc Natl Acad Sci USA 89: 10277–10281. 1438214 10.1073/pnas.89.21.10277 PMC50321

[22] Milne JC, Furlong D, Hanna PC, Wall JS, Collier RJ (1994) Anthrax protective antigen forms oligomers during intoxication of mammalian cells. J Biol Chem 269 (32): 20607–20612. 8051159

[23] Mogridge J, Cunningham K, Collier RJ (2002) Stoichiometry of anthrax toxin complexes. Biochemistry 41: 1079–1082. 11790132 10.1021/bi015860m

[24] Mogridge J, Cunningham K, Lacy DB, Mourez M, Collier RJ (2002) The lethal and edema factors of anthrax toxin bind only to oligomeric forms of the protective antigen. Proc Natl Acad Sci USA 99 (10): 7045–7048. 10.1073/pnas.052160199 PMC124525 11997437

[25] Friedlander AM (1986) Macrophages are sensitive to anthrax lethal toxin through an acid-dependent process. J Biol Chem 261: 7123–7126. 3711080

[26] Nanda A, Carson-Walter EB, Seaman S, Barber TD, Stampfl J, Singh S, et al (2004) TEM8 interacts with the cleaved C5 domain of collagen alpha-3(VI). Cancer Res 64: 817–820. 14871805 10.1158/0008-5472.can-03-2408

[27] Rmali KA, Al-Rawi MAA, Parr C, Puntis MCA, Jiang WG (2004) Upregulation of tumour endothelial marker-8 by interleukin-1-alpha induced angiogenesis. Int J Mol Med 14: 75–80. 15202019

[28] Rmali KA, Parr C, Puntis MCA, Jiang WG (2005) TEM-8 and tubule formation in endothelial cells, potential role of its vWA/TM domains. Biochem Biophys Res Commun 334: 231–238. 15993844 10.1016/j.bbrc.2005.06.085

[29] Carson-Walter EB, Watkins DN, Nanda A, Vogelstein B, Kinzler KW, et al (2001) Cell surface tumor endothelial markers are conserved in mice and humans. Cancer Res 61: 6649–6655. 11559528

[30] Premanandan C, Lairmore MD, Fernandez S, Phipps AJ (2006) Quantitative measurement of anthrax toxin receptor messenger RNA in primary mononuclear phagocytes. Microb Pathog 41: 193–198. 16854559 10.1016/j.micpath.2006.05.003

[31] Arora N, Klimpel KR, Singh Y, Leppla SH (1992) Fusions of anthrax toxin lethal factor to the ADP-ribosylation domain of Pseudomonas exotoxin A are potent cytotoxins which are translocated to the cytosol of mammalian cells. J Biol Chem 267: 15542–15548. 1639793

[32] Abrami L, Liu S, Cosson P, Leppla SH, van der Goot FG (2003) Anthrax toxin triggers endocytosis of its receptor via a lipid raft-mediated clathrin-dependent path. J Cell Biol 160: 321–328. 12551953 10.1083/jcb.200211018 PMC2172673

[33] Young JAT, Collier RJ (2007) Anthrax toxin: receptor binding, internalization, pore formation, and translocation. Annu Rev Biochem 76: 243–65. 17335404 10.1146/annurev.biochem.75.103004.142728

[34] Abrami L, Bischofberger B, Kunz R, Groux F, van der Goot FG (2010) Endocytosis of the anthrax toxin is mediated by clathrin, actin and unconventional adaptors. PLoS Pathog 6: e1000792. 20221438 10.1371/journal.ppat.1000792 PMC2832758

[35] Kintzer AF, Thoren KL, Sterling HJ, Dong KC, Feld GK, et al (2009) The protective antigen component of anthrax toxin forms functional octameric complexes. J Mol Biol 392(3): 614–629. 19627991 10.1016/j.jmb.2009.07.037 PMC2742380

[36] Chaudry GJ, Moayeri M, Liu S, Leppla SH (2002) Quickening the pace of anthrax research: three advances point towards possible therapies. Trends Microbiol: 10, 58–62. 11827799 10.1016/s0966-842x(01)02294-6

[37] Bonuccelli G, Sotgia F, Frank PG, Williams TM, De Almeida CJ, et al (2005) ATR/TEM8 is highly expressed in epithelial cells lining *Bacillus anthracis’* three sites of entry: implications for the pathogenesis of anthrax infection. Am J Physiol Cell Physiol 288(6): C1402–1410. 15689409 10.1152/ajpcell.00582.2004

[38] De Preter K, Pattyn F, Berx G, Strumane K, Menten B, et al (2004) Combined subtractive cDNA cloning and array CGH: an efficient approach for identification of overexpressed genes in DNA amplicons. BMC Genomics 5: 11–24. 15018647 10.1186/1471-2164-5-11 PMC365025

[39] Oberthuer A, Skowron M, Spitz R, Kahlert Y, Westermann F, et al (2005) Characterization of a complex genomic alteration on chromosome 2p that leads to four alternatively spliced fusion transcripts in the neuroblastoma cell lines IMR-5, IMR-5/75, and IMR-32. Gene 363: 41–50. 16216448 10.1016/j.gene.2005.07.038

[40] Liu S, Crown D, Miller-Randolph S, Moayeri M, Wang H, et al (2009) Capillary morphogenesis protein-2 is the major receptor mediating anthrax toxin lethality in vivo. Proc Natl Acad Sci 106 (30): 12224–12229. 10.1073/pnas.0905409106 PMC2718377 19617532

[41] Reeves CV, Dufraine J, Young JA, Kitajewski J (2010) Anthrax toxin receptor 2 is expressed in murine and tumor vasculature and functions in endothelial proliferation and morphogenesis. Oncogene 29 (6): 789–801. 10.1038/onc.2009.383 PMC2939496 19901963

[42] Hotchkiss KA, Basile CM, Spring SC, Bonuccelli G, Lisanti MP, et al (2005) TEM8 expression stimulates endothelial cell adhesion and migration by regulating cell-matrix interactions on collagen. Exp Cell Res 305: 133–144. 15777794 10.1016/j.yexcr.2004.12.025

[43] Wei W, Lu Q, Chaudry GJ, Leppla SH, Cohen SN (2006) The LDL receptor-related protein LRP6 mediates internalization and lethality of anthrax toxin. Cell 124: 1119–1121. 16564009 10.1016/j.cell.2005.12.045

[44] Abrami L, Leppla SH, van der Goot FG (2006) Receptor palmitoylation and ubiquitination regulate anthrax toxin endocytosis. J Cell Biol 172: 309–20. 16401723 10.1083/jcb.200507067 PMC2063559

[45] Go MY, Chow EM, Mogridge J (2009) The cytoplasmic domain of anthrax toxin receptor 1 affects binding of the protective antigen. Infect Immun 77 (1): 52–59. 10.1128/IAI.01073-08 PMC2612287 18936178

[46] Yang MY, Chaudhary A, Seaman S, Dunty J, Stevens J, et al (2010) The cell surface structure of tumor endothelial marker 8 (TEM8) is regulated by the actin cytoskeleton. Biochim Biophys Acta 1813: 39–49. 21129411 10.1016/j.bbamcr.2010.11.013 PMC3014418

