# Correction: Broad Expression Analysis of Human ANTXR1/TEM8 Transcripts Reveals Differential Expression and Novel Splizce Variants

**DOI:** 10.1371/annotation/cebf633f-19e7-496b-b370-c0f1b1aea888

**Published:** 2012-10-22

**Authors:** Micaela Vargas, Raghavendra Karamsetty, Stephen H. Leppla, G. Jilani Chaudry

The word "Splice" is misspelled in the article title. The correct title is: Broad Expression Analysis of Human ANTXR1/TEM8 Transcripts Reveals Differential Expression and Novel Splice Variants.

The correct citation is: Vargas M, Karamsetty R, Leppla SH, Chaudry GJ (2012) Broad Expression Analysis of Human ANTXR1/TEM8 Transcripts Reveals Differential Expression and Novel Splice Variants. PLoS ONE 7(8): e43174. doi:10.1371/journal.pone.0043174 

